# Superhydrophobic Ni-Reduced Graphene Oxide Hybrid Coatings with Quasi-Periodic Spike Structures

**DOI:** 10.3390/nano12030314

**Published:** 2022-01-19

**Authors:** Ayi Bahtiar, Mila Sri Hardiati, Ferry Faizal, Vanitha Muthukannan, Camellia Panatarani, I Made Joni

**Affiliations:** 1 Department of Physics, Faculty of Mathematics and Natural Sciences, Universitas Padjadjaran, Jalan Raya Bandung-Sumedang KM 21, Jatinangor, Sumedang 45363, West Java, Indonesia; mila.srihardiati@gmail.com (M.S.H.); ferry.faizal@unpad.ac.id (F.F.); c.panatarani@phys.unpad.ac.id (C.P.); imadejoni@phys.unpad.ac.id (I.M.J.); 2 Functional Nano Powder University Center of Excellence (FiNder U CoE), Universitas Padjadjaran, Jalan Raya Bandung-Sumedang KM 21, Jatinangor, Sumedang 45363, West Java, Indonesia; vanithachel@gmail.com

**Keywords:** reduced graphene oxide, Ni-reduced graphene oxide, graphene, oxidation, microwave, superhydrophobic

## Abstract

Recently, sophisticated technologies are applied to design a certain surface nature that can have superhydrophobic properties. Thus, a simple spray technique was introduced to prepare a superhydrophobic surface using rGO with Ni-S system (rGO-Ni) by using NiSO_4_ catalyst under microwave irradiation at various reaction times of 5, 10, 20, and 30 min. The GO reduction was conducted at a fixed Ar/H_2_ ratio, a flow rate of 0.4 L/min, microwave power of 720 W, and a mass of 0.5 g. GO powder with nickel sulfate catalyst was treated under Ar/H_2_ (4:1) mixture for GO reduction, where Ar and H_2_ were expected to prevent the rebinding of oxygen released from GO. The result of XRD and Raman measurement confirms that rGO-Ni prepared at reaction time 20 min exhibit the highest reduction of GO and the presence of various Ni-S crystal structures such as NiS, NiS_2_, Ni_3_S_2_, and Ni_3_S_4_ due to decomposition of NiSO_4_. The rGO-Ni coating performance shows superhydrophobic nature with a contact angle of 150.1°. The AFM images show that the addition of nickel to rGO produces a quasi-periodic spike structure, which increases the superhydrophobicity of the r-GO-Ni coated glass with a contact angle of 152.6°. It is emphasized that the proposed simple spray coating using rGO-Ni provides a more favorable option for industry application in obtaining superhydrophobic surfaces.

## 1. Introduction

Graphene and its derivative compounds such as graphene oxide (GO) and reduced graphene oxide (rGO) recently have become promising materials that are entitled to be developed because of their properties, such as high electrical conductivity, thermal conductivity, surface area, and tensile strength [1,2,3,4]. These properties enable graphene to be used for various applications such as photovoltaic cells, sensors, chemical energy storage devices, transistors, transparent electrodes, and coatings. Graphene and graphene-related materials have become increasing research interests due to many remarkable features that make them suitable for a passive-layer formation that protects metals from oxidation and corrosion [5,6,7,8]. Graphene and its related materials can also be applied as coating glass for self-cleaning glass that causes a superhydrophobic surface of the glass [9,10]. A superhydrophobic surface can be characterized by its water contact angle in the range of 150° < θ < 180°; therefore, water droplets can roll on the surface and take away the dirt sticking on the surface effectively.

Graphene can be produced by exfoliation of graphite, epitaxial growth, chemical vapor deposition (CVD), and reduction of graphene oxide [11,12]. The reduction of GO that formed reduced graphene oxide (rGO) is the most popular method to produce graphene because of its low production costs and a larger quantity of the material compared to other methods [11]. Graphene oxide is the exfoliated graphite that is treated in an oxygen-rich environment and oxidized to intersperse the carbon layers with oxygen molecules that have reactive functional groups such as hydroxyl, epoxy, and carboxyl groups [3,12,13]. GO is reduced by various methods to separate layers of carbon into a material that resembles graphene called rGO [13].

Thermal reduction is one of the reduction methods that is often used by carrying out the reaction at high temperatures that reach above 2000 °C [14,15,16]. As an alternative, Zou et al. (2003) [17] and Chu and Li (2006) [18] have tried to use several unconventional heating sources to reduce temperatures, including microwave irradiation (MWI). The main advantage of microwave irradiation over conventional heating methods is that heating occurs uniformly and rapidly. Reduced graphene oxide (rGO) can be easily obtained within one minute by treatment under microwave irradiation. Aside from temperature, the heating atmosphere is also important for the reduction process of GO, and oxygen increases dramatically at high temperatures. Therefore, oxygen must be released during heating. Thermal reduction is usually carried out in a vacuum, an inert atmosphere, or in presence of reducing gases. Argon (Ar), known as an inert gas, can prevent released oxygen back to the material. Reducing gases such as hydrogen (H_2_) can react with the remaining oxygen in the atmosphere, and they flow along with the gas flow. The ability of H_2_ to reduce O_2_ is adequate to make the reduction of GO and can be finished at relatively low temperatures in the H_2_ atmosphere [11].

Khai et al. (2013) [19] warmed GO sheets at a high temperature of 1100 °C for 30 min on a system with gas (Ar + H_2_) flowing at a constant speed of 100 sccm, to reduce humidity and temperature of the system. Chen et al. (2010) [20] also reduced GO by microwave hydrothermal with a mixture of N, N-dimethylacetamide, and water. The power of the microwave is 800 W, and rGO with a conductivity of 200 S/m is produced in a few minutes. Recently, we have successfully synthesized rGO by microwave-assisted methods within the Ar/H_2_ gas flow rate of 0.4 L/min and microwave power of 720 Watt provide optimum oxygen group reduction [21].

Superhydrophobic surfaces with water contact angles (CA) > 150° have increased attention due to their numerous potential applications such as anti-icing, self-cleaning, oil–water separation, and anticorrosion [22,23,24]. Reduced graphene oxide (rGO) is a suitable candidate as superhydrophobic material, due to its hydrophobicity in nature, its large specific surface area, abrasion resistance, and excellent conductivity. Various methods such as sol–gel, 3D printing, chemical etching, chemical vapor deposition, electrospinning, chemical bath deposition, polymer grafting, and self-assembly have been used to prepare superhydrophobic surfaces [25,26]. Another group also reports on the engineering of the material to improve the hydrophobicity of the surface such as using polydimethylsiloxane as a transparent agent [27]. However, most of these methods are not favorable for industrial application due to their complexity and time-consuming procedures. Finding a simple method for producing superhydrophobic surfaces is, therefore, highly needed for large-scale applications. Recently, Esmailzadeh et al. used the electrodeposition technique to prepare a thin nickel layer consisting of micro-cones structure on a copper substrate, producing a superhydrophobic surface with water CA of 155° [28]. The composite of rGO and nickel, therefore, can be very interesting to be studied for preparing superhydrophobic surfaces with a high value of CA. Ding et al. prepared a superhydrophobic rGO/Ni composite coating on mild steel with water CA 160.4° using the electrodeposition technique [29]. However, the substrate could not be covered completely by the rGO/nickel composite particles after the deposition process. Recently, Bai and Zhang prepared a novel rGO/Ni composite coating with pinecone-like micro/nanostructures on a stainless steel substrate using an electrodeposition method combining Ni pre-deposition and an elevated current assistant approach [30]. The coating showed a self-cleaning effect and superhydrophobicity, with a CA of 162.7°. Another report by Schneider et al. showed that a regular micro- and nanocone structure induce superhydrophobicity of the surface [31]. Therefore, producing periodic micro- and nanostructure or spike structures on the coated surface will produce a superhydrophobic layer with a high value of CA.

Therefore, the aims of the present study are to study the properties of rGO and rGO with Ni-S system using NiSO_4_ catalyst (rGO-Ni) and evaluate their hydrophobicity performance of a composite coating in comparison with graphite and without any filler. The spray technique on the preparation of superhydrophobic surface with rGO-Ni composite is a novel approach to provide more favorable for industry, compared with other sophisticated available techniques to the engineering of the surface morphology. In this study, we prepared an rGO-Ni coated layer from GO reduction synthesized by the microwave-assisted method under the mixture of Ar and H_2_ gases at various reaction times.

## 2. Materials and Methods

### 2.1. Preparation of Graphene Oxide

The chemicals used in the preparation of graphene oxide were graphite (Merck), sodium nitrate (NaNO_3_), sulfuric acid (H_2_SO_4_), potassium permanganate (KMnO_4_), concentrated hydrochloric acid (HCl), and hydrogen peroxide (H_2_O_2_). The graphite was purchased from Merck Chemicals, Jakarta, Indonesia, and the graphene oxide (GO) was prepared following the modified Hummers method [32]. Two grams of graphite powder and one gram of sodium nitrate were mixed in 45 mL of sulfuric acid, and the mixture was continuously stirred in an Erlenmeyer flask stored within the ice bath (0–5 °C) for 30 min. Then, five grams of potassium permanganate was prepared and added to the mixture by a small amount stepwise. The precaution was necessary when introducing the potassium permanganate to maintain a temperature less than 20 °C. The mixture was stirred once more for 30; then, the ice bath was substituted with a silicon oil bath. The sample was stirred at 35 °C for one hour. The mixture was then subjected to dilution with slowly added 45 mL of distilled water. Consequently, the temperature rapidly rose to 98 °C while keeping stirring for another 40 min. Additional dilution was carried out for 200 mL of distilled water and 25 mL of hydrogen peroxide while keeping stirring for 30 min. Finally, 10 mL of HCl solution in deionized water was used to clean the sample and continuously washed several times with distilled water. The sample was centrifuged to separate the substances. GO was dried in a drying cabinet to obtain the GO powder.

### 2.2. Mixing with Nickel Sulfate

An experiment by Joni et al. (2008) [33] shows that GO synthesized from graphitization with a nickel sulfate catalyst is better than GO synthesized from commercial graphite. Therefore, in this experiment, GO powder was mixed with nickel sulfate. The procedure for mixing GO with NiSO_4_ catalyst was obtained by dissolving 1.578 g of NiSO_4_ with 10 mL of distilled water and stirring for 1 h until completely dissolved. Then, one gram of GO was added into the NiSO_4_ solution and stirred for 4 h. The residue was filtered and dried in an oven for 5 h at 65–70 °C [33]. After the drying process, GO powder with a NiSO_4_ catalyst was produced. This GO sample was then subjected to a reduction process using microwave irradiation.

### 2.3. Reduction of GO

**Figure 1:**
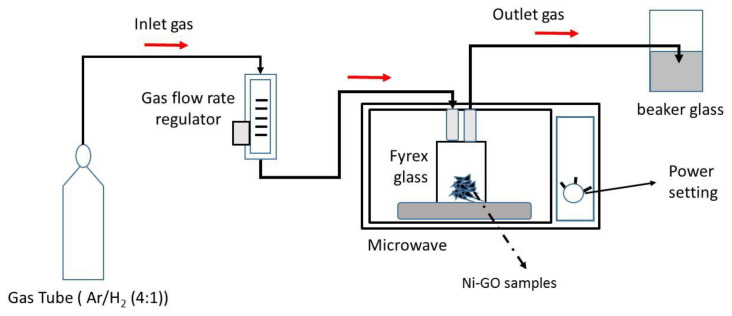
Schematic experimental setup of GO microwave irradiation.

The GO reduction process was carried out using the microwave irradiation method, with equipment arranged as shown in Figure 1, referring to the best-optimized reduction process from our previous study [21]. The sample with a mass of 0.5 g was added into the separate glass, which was tightly closed and kept in the microwave oven. Ar/H_2_ gas mixture was passed through the glass by means of the inlet pipe and exited through the outlet pipe with a flow rate of 0.4 L/min. An indication of gas discharge is the presence of air bubbles in the water placed on a beaker. Furthermore, the microwave oven was turned on, by setting the power of 720 W [34]. The reduction process was conducted in various reaction times 1, 5, 10, 20, and 30 min.

### 2.4. Preparation of GO Layer

For this step, 0.1 g (5%) GO was added into 1.3 g (65%) thinner solution (mixing of butyl acetate (BA) and xylene with ratio 1:1) and then stirred for 30 min. Afterward, 0.6 g (30%) of binder (TPA resin) was added to the solution and was stirred for 30 min. The GO layer was prepared by spraying the solution onto the preparatory glass using a Meiji air paintbrush with a distance of about 30 cm from the surface of the glass. Then, it was dried in an oven at 350 °C for 30 min. A similar procedure was carried out for the preparation of graphite, rGO, and rGO-Ni layers. For contact angle testing, 5 µL of deionized water solution was dropped onto the layers. A glass coated by paint was used as a control for the comparison of contact angle testing and AFM measurement.

### 2.5. Characterization

The synthesized GO by modified Hummer’s method, as well as rGO and rGO-Ni by microwave irradiation methods, were characterized by Fourier-transform infrared spectroscopy (FTIR, Nicolet Is5 Thermo, Toronto, ON, Canada), X-ray diffraction analysis (XRD, PANalytical X’Pert PRO PW3040/x0, Almelo, Netherlands), scanning electron spectroscopy–energy dispersive spectrometer (SEM–EDS, SU3500 Hitachi -EDAX TEAM, Tokyo, Japan), Raman spectroscopy (XploRA™ PLUS – HORIBA, Kyoto, Japan) contact angle testing (laboratorymade), and atomic force microscopy (AFM, Park System XE 100, Suwon, Korea).

## 3. Results and Discussion

### 3.1. FTIR Analysis

**Figure 2:**
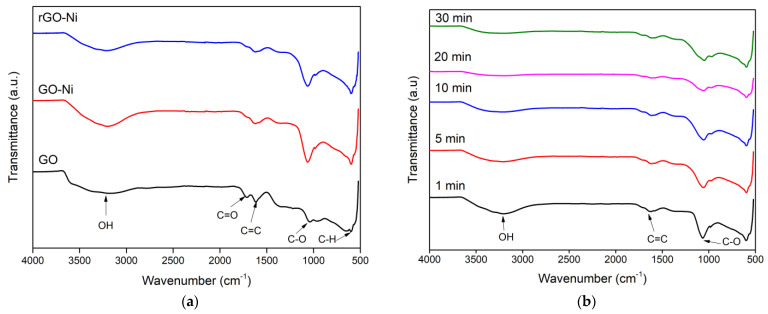
The FTIR spectra of (**a**) GO and GO−Ni and rGO−Ni prepared with 0.5 g GO at gas flow rate 0.4 L/min, one minute reaction time, and with a microwave power of 720 W and (**b**) rGO−Ni prepared at 720 W with a variation of reaction times.

FTIR spectra of GO before and after mixing with the NiSO_4_ catalyst or GO-Ni and rGO-Ni prepared with 0.5 g GO, flowrate 0.4 L/min, microwave power of 720 W, and one-minute reaction are shown in Figure 2a. The peak at 1060 and at 1625 cm^−1^ are correspondingly identified as C-O and C=C bonds. The C-O bond indicated the existence of oxygen functional groups on the surface of GO. The C=C function group is the elementary structure of GO or rGO, which is bound together and forms a hexagonal structure where the double bond is a covalent bond formed from sp^3^ hybridization into sp^2^ and is difficult to be disrupted because of its higher bond energy. A peak at 1720 cm^−1^ in the GO spectrum relates to the C=O bonds. A broad peak is observed around 2900–3650 cm^−1^ due to the O-H stretching of the H_2_O molecule. The spectra assigned for oxygen groups in GO-Ni show more visibly than GO due to the enhanced oxidation process by NiSO_4_ catalyst on the surface of the synthesized GO. The spectrum of rGO-Ni shows oxygen-containing groups, i.e., OH and C=O lines are decreased, compared with GO, which indicated that the oxygen-containing group was reduced. This indicated that our proposed microwave irradiation methods for the mixture of GO and NiSO_4_ successfully reduced the oxygen functional groups [35].

In order to find the optimum reaction time, the reaction times were varied for 5 min, 10 min, 20 min, and 30 min, with fixed parameters of mass of 0.5 g, a gas flow rate of 0.4 L/min, and microwave power of 80P (720 W). FTIR results from time variation experiments can be seen in Figure 2b, which shows a significant difference. It can be seen that at the time of 20 min, the peaks of the oxygen-containing groups on the O-H and C=O vary dramatically to resemble almost a straight line. This indicates that the reduction process of 20 min is the best reaction time because the oxygen-containing groups in the sample have reduced significantly. The graph also shows that the peak for C=C at a wavenumber of 1605 cm^−1^ is still visible. When GO is irradiated by microwave at certain times, the oxygen bond in the GO will be released and turn into ions and subsequently released along with the gas flow. If the irradiation time of GO is prolonged, more oxygen bonds are released. Thus, the success of reduction to prevent oxygen rebinding into GO depends on the ratio of the amount of released oxygen and H_2_ and expel as H_2_O. After reaction times of more than 20 min, the hydrogen gas may react with increasing oxygen released from GO to form O-H binding on the GO surface, as shown in Figure 2b (30 min).

### 3.2. XRD Analysis

**Figure 3:**
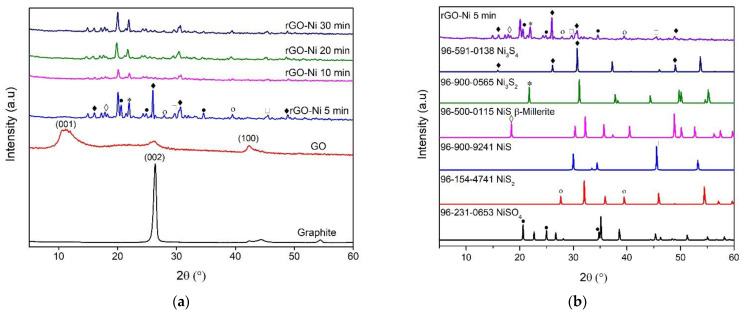
XRD pattern of (**a**) graphite, GO, and rGO-Ni with various reaction times, and (**b**) rGO-Ni with reaction times 5 min in comparison with various Ni-S crystal structures.

XRD patterns of graphite, GO, and rGO-Ni with various reaction times are shown in Figure 3a. The XRD pattern of pure graphite reveals an intense peak around 26°, which corresponds to the crystal plane (002). The XRD pattern of GO illustrates three peaks at 11°, 26°, and 42.5°, corresponding to the peak of the crystal plane (001), (002), and (100), respectively. The diffraction peak at 2θ = 11° is caused by graphitic oxidation, according to the GO peak, which shows the intercalation of various functional groups after oxidation of graphite. Graphite was successfully oxidized to GO, which shows a characteristic peak at 2θ = 11°. The graphite peak still exists at 26°, and the graphite group can be adjusted by varying the concentration of the material used in the oxidation process and obtaining the conversion of graphite to GO completely. The peak at 2θ = 42.2° with small intensity originates from a non-oxidized graphitic group [34]. The absence of a peak around 10–11° in the XRD pattern of rGO-Ni confirms the reduction of GO to rGO.

The crystal structure of rGO-Ni varies in reaction times of 5, 10, 20, and 30 min exhibited various Ni-S crystal structures (Figure 3a). In order to identify the crystal phase transformation during microwave irradiation due to NiSO_4_ decomposition, the XRD of r-GO-Ni with reaction times 5 min is compared with possible XRD of the Ni-S system (Figure 3b). The XRD pattern at a reaction time of 5 min clearly appears to various Ni-S crystal structures such as NiS, NiS_2_, Ni_3_S_2_, and Ni_3_S_4_ due to the decomposition of NiSO_4_. The XRD peak for NiSO_4_ is vanished, indicating decomposition occurred during microwave irradiation (Figure 3a). At reaction time 5 min, two remarkable XRD peaks at 26° and 31.7° are observed, indicating the initial crystal phase formation of Ni_3_S_4_. After reaction time elapses for 10, 20, and 30 min, the peak at 26° is disappeared to form a Ni_3_S_4_ crystal structure. It is observed that all Ni-S systems such as NiS, NiS_2_, Ni_3_S_2_, and Ni_3_S_4_ are still present at reaction times 10, 20, and 30 min. In contrast, the highest peak appears at 20° at reaction times 10, 20, and 30 min, representing the crystal system of the rGO decorated N-S system [35].

### 3.3. Raman Spectroscopy

**Figure 4:**
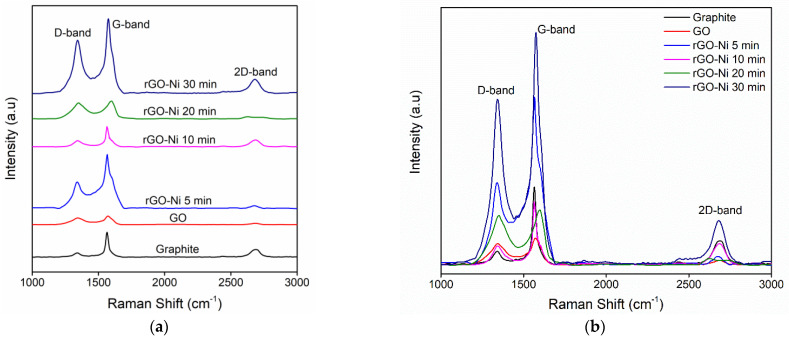
(**a**) Raman shift of graphite (G), GO, and rGO-Ni at various reaction times, and (**b**) their intensities comparison of Raman shift.

Raman spectroscopy is one of the powerful techniques used to characterize carbon-based materials, as it is non-destructive, fast, and has high resolution. The structural and electronic information of the conjugated and double-bonded carbon–carbon leads to high intense peaks in Raman spectroscopy [11,19,36]. Figure 4 shows the Raman shift of graphite, GO, and rGO-Ni with reaction time variation.

Graphite has the G band at 1565 cm^−1^ and the D band at 1330 cm^−1^. The G band correlates to the in-phase C-C stretching vibration of the graphite, and the D band represents the disordered band caused by the graphite edges. The D band is present in all defective graphene samples, and therefore, it is a favorable measure of the quality of the carbon produced. Raman spectra of GO show the G band with decreased intensity caused by the disordered of graphite lattice, which was introduced upon oxidation. The reduction of GO restores the position of the G band to almost the same position as graphite, indicating a considerable restoration of the graphitic lattice. The overall intensity of Raman spectra increases after the reduction treatment, which substantiates the increased carbon-to-oxygen ratio, owing to the NiSO_4_ catalyst used during the reduction process. GO reduction induces deviations in its structure due to the removal of oxygen and some carbon atoms [37,38]. The intensity ratio of the D band to G band (I_D_/I_G_) is commonly used to measure the degree of disordered carbon, as expressed by the sp^3^/sp^2^ carbon ratio. The degradation of crystallinity of graphitic materials is indicated by an increased value of I_D_/I_G_ [19].

**Figure 5:**
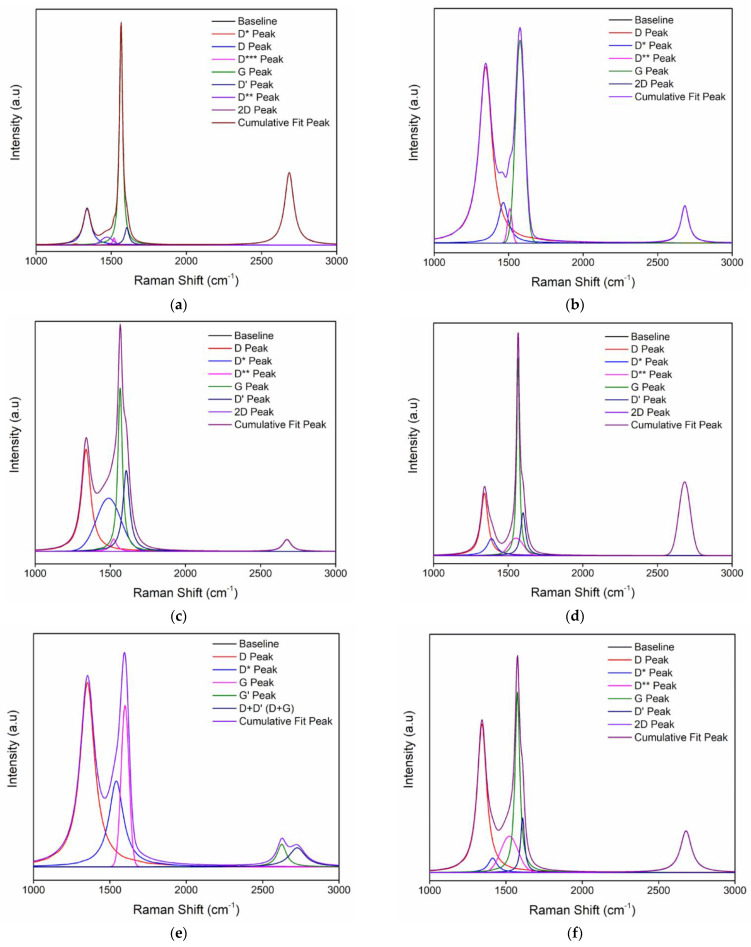
Multiple peak-fit of Raman spectra of (**a**) graphite, (**b**) GO, and rGO−Ni for the reaction time (**c**) 5 min, (**d**) 10 min, (**e**) 20 min, and (**f**) 30 min.

Figure 5 shows the multiple-peak-fit Raman spectra of graphite, GO, and rGO-Ni for different reaction times. Figure 5a shows the multiple peak-fit of Raman spectrum of graphite that carried on the D and G bands. A total of seven peaks could be fit, as shown in Table 1. The peak at 1339 cm^−1^ shows the D band, and the peak at 1565 cm^−1^ shows the G band. The peak at 1604 cm^−1^ is the D’ band that makes the G band broadened. There are three additional peaks (D*, D**, D***) found at 1263 cm^−1^, 1471 cm^−1,^ and 1518 cm^−1^, respectively. The 2D band is found at 2684 cm^−1^.

Figure 5b–f shows the multiple peak-fit of Raman spectrum of GO and rGO that carried on the D and G bands, and their fitting is presented in Table 2. Figure 5b shows the multiple peak-fit for the Raman spectrum of GO with D and G bands. A total of five peaks were fit, as depicted in the figure. The peak at 1345 cm^−1^ shows the D band, and the peak at 1576 cm^−1^ corresponds to the G band. There are two additional peaks (D*, D**) found at 1464 cm^−1^ and 1507 cm^−1^, and other peak at 2682 cm^−1^ represents the 2D band.

Figure 5c shows the multiple peak-fit of Raman spectrum of rGO-Ni for 5 min of reaction time. A total of six peaks could be fit as shown. The peak at 1338 cm^−1^ reveals the D band, and the peak at 1565 cm^−1^ signifies the G band. There are three additional peaks (D*and D**) at 1488 cm^−1^ and 1522 cm^−1^. The other peak at 1604 cm^−1^ relates to D’ band that makes the G band broadened. The 2D band is found at 2682 cm^−1^.

Figure 5d shows the multiple peak-fit Raman spectrum of rGO-Ni for 10 min of reaction time for the D and G bands. A total of six peaks could be fitted, as shown in the figure. The peak at 1341 cm^−1^ and at 1566 cm^−1^ corresponds to the D and G band, respectively. There are three additional peaks (D*and D**) occurring at 1391 cm^−1^ and 1546 cm^−1^. The peak at 1599 cm^−1^ is the D’ band that makes the G band broadened. The 2D band is found at 2682 cm^−1^.

Figure 5e shows the multiple peak-fit of Raman spectrum of rGO-Ni for 20 min of reaction time for D and G bands. A total of five peaks could be fitted as shown. The peak centered at 1341 cm^−1^ represents the D band, and the peak centered at 1566 cm^−1^ corresponds to the G band. There are three additional peaks (D*and D**) occurring at 1391 cm^−1^ and 1546 cm^−1^. The peak at 1599 cm^−1^ is the D’ band that leads to the broadening of the G band. The shape of the 2D band on multiple peak-fit of rGO-Ni for 20 min of reaction time has two peaks rather than a single Lorentzian. The G’ peak is found at 2625 cm^−1^, and the D + D’ (D + G) peak is found at 2726 cm^−1^.

Figure 5f shows the multiple peak-fit Raman spectrum of rGO-Ni for the reaction time of 30 min. A total of six peaks could be fitted as shown. The peak at 1341 cm^−1^ indicates the presence of the D band, and the peak at 1566 cm^−1^ discloses the G band. There are three additional peaks (D*and D**) that are found at 1391 cm^−1^ and 1546 cm^−1^. The peak at 1599 cm^−1^ is the D’ band that makes the G band broadened. The shape of the 2D band on multiple peak-fit of rGO-Ni for 20 min of reaction time has two peak profiles rather than a single Lorentzian. The G’ peak is found at 2625 cm^−1^, and the D + D’ (D + G) peak is found at 2726 cm^−1^.

In graphite and rGO, the G band consists of a superposition of two peaks—namely, the G and D’ peaks. The intensity of the D’ peak in graphite is proportional to the crystallite size and correlates to the number of defects. For rGO, which has comparatively high defect densities, the D’ peak is more intense and thus contributes significantly to the graphene peak. The D’ peak of graphene samples is diverse from the G peak, but the properties and spectral signature of defective graphene do not close to those of even highly reduced GO [34,36]. In contrast, the highest intensity ratio of D band to G band (I_D_/I_G_) is obtained from the reaction times 20 min indicated that the degree of oxygen released is higher, compared with other reaction times (Table 2). This result supports the spectra analysis that also finds at 20 min is the optimal reaction time.

The half-width at half maximum above the graphene centroid position is larger for many GO samples than expected, illustrating that the G band is accompanied by an extra peak at slightly higher energy. By increasing the density of defects, D’ modes occur at lower energy around 1580 cm^−1^, which would theoretically make it appear at lower energy than the G peak. The background of this energy shift of the D’ mode remains unclear but may depend on the second-nearest force constants. The influence of such a shift on the position and intensity of graphene has not been explored [39]. The origin of the D*, D**, and D*** bands is not clearly understood. Some reports suggest that the D* peak represents sp^3^ rich phase of disordered amorphous carbons. The D** peak can be caused by the contributions of phonon density of states in finite-size graphitic crystals or by C-H vibrations in hydrogenated carbons [40].

### 3.4. SEM–EDS

**Figure 6:**
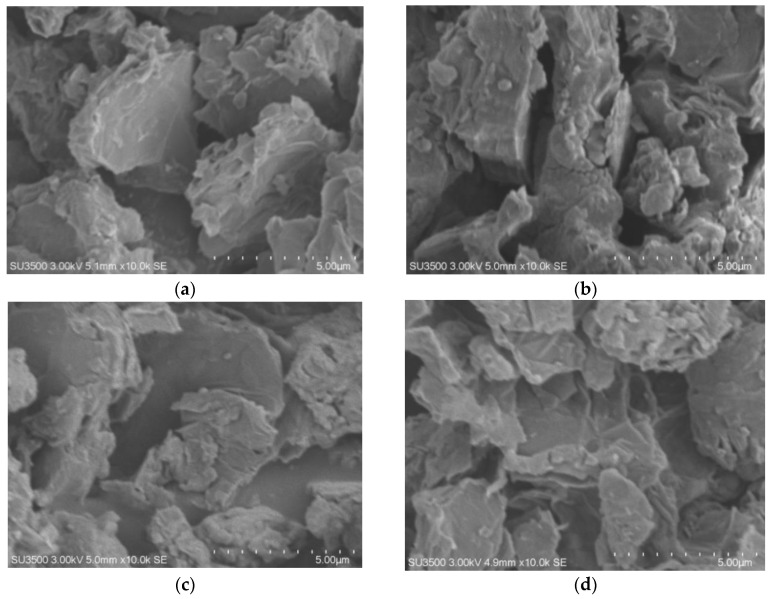
SEM Morphologies of rGO-Ni for (**a**) 5 min, (**b**) 10 min, (**c**) 20 min, and (**d**) 30 min reaction time.

SEM is used to determine the surface morphology of the material. By characterizing the samples with SEM, the material topography, grain size, and composition of a sample (EDS) can be evidently observed. SEM characterization was carried out for the samples of rGO-Ni, with varying reaction times, and the SEM image of rGO is depicted in Figure 6. The SEM image reveals sheet-like morphology for the various reaction times, and not much difference is observed. The elemental composition of the rGO-Ni with time variations can be seen in Table 3.

While there is not much information obtained from the SEM micrograph of rGO-Ni for various reaction times, EDS analysis discloses important information about the C/O ratio. EDS is one of the ways to determine the success of the oxygen reduction process by observing the difference in the atomic weight of elements present in rGO-Ni. The C/O ratio is high for 10 min and 20 min of reaction time, and the percentage of oxygen is relatively low for both the reaction times. Based on other studies, 20 min reaction was considered as optimum. Based on the percentage of composition of the majority of the atoms in Table 3, it can be observed that the carbon atom represents the main constituent material, and the presence of oxygen atom indicates GO was not completely oxidized. It should be noted that the mass percentage of Ni and S present is due to the use of nickel sulfate as a catalyst for the reduction process using microwave irradiation.

### 3.5. Contact Angle

**Figure 7:**
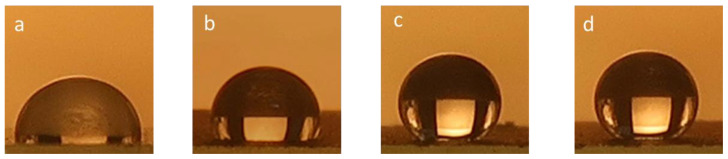
Photographs of DI water drop onto (**a**) painted glass, (**b**) graphite coated on painted glass, (**c**) rGO coated on painted glass, and (**d**) rGO-Ni coated on painted glass. The contact angle values of (**a**–**d**) are 94.8°, 118.7°, 150.1°, and 152.6°, respectively.

Figure 7 shows a photograph of the drop of deionized water solution on the graphite, rGO, and rGO-Ni layers deposited onto paint-coated glass. A photograph of a glass-coated only by paint is also shown for comparison. The contact angle of a paint-coated glass is 94.8°, which shows the hydrophobic surface. The contact angle is increased to 118.7°, 150.1°, and 152.6° when the surface was coated with graphite, rGO, and rGO-Ni, respectively. These results show that the surface changes from hydrophobic to superhydrophobic when it was coated by rGO and rGO-Ni. Moreover, the addition of nickel improves the superhydrophobicity of the painted glass, which might be caused by the surface roughness and hierarchical nanostructures or periodic spike structure of the surface.

### 3.6. AFM

**Figure 8:**
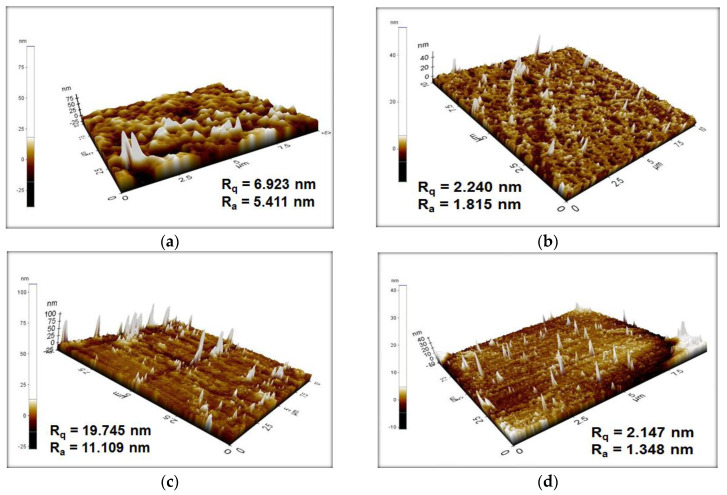
AFM images of the film surface (**a**) painted glass, (**b**) graphite coated on painted glass, (**c**) rGO coated on painted glass, and (**d**) rGO−Ni coated on painted glass. The surface roughness values of all films are inserted.

Surface images of painted glass coated by graphite, rGO, rGO-Ni, and painted glass only are shown in Figure 8. We scanned the surface of the films to obtain the surface roughness, indicated by the value of R_q_ (root mean square roughness) and Ra (average roughness). R_q_ is the root of the average square of the surface roughness level, while the value of Ra is the arithmetic mean of the surface height. The values were obtained from the difference in height at several positions, compared with the arithmetic average from the surface [41]. The R_q_ values of painted samples without binder, coated by graphite, rGO, and rGO-Ni are 6.923 nm, 2.240 nm, 19.745 nm, and 2.147 nm, respectively. Meanwhile, the values of Ra are 5.411 nm, 1.815 nm, 11.109 nm, and 1.348 nm for painted samples without binder, coated by graphite, rGO, and rGO-Ni, respectively.

Surface roughness-induced superhydrophobicity has been intensively studied in terms of the lotus effect, where the hydrophobicity properties of the surface can be increased by increasing the roughness of the surface [42,43]. Lotus leaves have hierarchical micro/nanoscale structures, where water on such a surface forms a spherical droplet and significantly reduces the contact area and interaction between water and the surface or adhesion to the surface [44]. Both theoretical models and experimental studies have been carried out to determine the effect of surface roughness on surface wettability, which is represented by contact angle value [25,45,46,47]. Wenzel and Cassie–Baxter models are mostly used to describe and predict the contact angle on rough surfaces. The Wenzel model uses a homogeneous interface that considers a water droplet that seeps in between the irregularities of a rough surface. The Cassie–Baxter model considers a water droplet that sits on top of the irregularities and forms a layer trapped between the irregularities beneath it, leading to a composite solid–liquid–air interface. In the Wenzel model, the contact angle of the rough surface increases with increasing surface roughness, which is defined as the ratio of the solid–liquid area to its projection on a flat plane [48]. In the Cassie–Baxter model, the contact angle depends on the surface roughness, but also on the fractional flat geometric area of the liquid–air interfaces under the droplet or air pockets. These air pockets underneath the liquid will reduce the contact area between water/liquid and surface, therefore increasing the contact angle. Periodic structures such as pillars or cones or spikes in micro- or nanometers size are commonly fabricated on the surface to improve the CA [49].

The AFM image of the painted glass in Figure 8a shows a rough surface, which causes the hydrophobic surface with the CA value of 94.8°, as described both using Wenzel and Cassie–Baxter models. Graphite deposition on painted glass produces nanostructures or pillars or spikes with a height of less than 40 nm, which are almost uniformly distributed, as shown in Figure 8b. These nano-spikes increases numbers of air pocket formation that reduces the contact area between water and surface, as described by the Cassie–Baxter model. As a consequence, the CA value is increased to 118.7°. The height of these spikes is increased when rGO is deposited on the painted glass, as shown in the AFM image in Figure 8c. As a result, the surface becomes superhydrophobic, as indicated by the CA of 150.1°. Nickel doping on rGO (rGO-Ni) produces spikes that are more evenly distributed or a periodic spike structure on the surface, as shown in Figure 8d, thereby increasing the contact angle value to 152.6°. Our study shows that the superhydrophobic surface of the sample can be prepared by deposition of nickel-doped rGO (rGO-Ni) using a simple spray technique, which is a more practical use for large-area coating of the surface and, therefore, more favorable for the industry.

## 4. Conclusions

In this study, GO was treated with nickel sulfate catalyst under Ar/H_2_, in the ratio of 4:1, by microwave irradiation method. The role of Ar and H_2_ is to bind the oxygen within the system. FTIR analysis was performed to determine the reduction of GO to rGO, using nickel sulfate as a catalyst. Based on the analysis, the reaction time of 20 min in Ar/H_2_ atmosphere with a flow rate of 0.4 L/min, microwave power of 720 W, and the sample mass of 0.5 g was optimal. The reduction of GO to rGO was also reiterated by XRD studies. Raman spectroscopic analysis and peak fitting were performed for graphite, GO, rGO, and rGO-Ni with various reaction times (5, 10, 15, 20, and 30 min). The SEM analysis reveals sheet-like morphology for the prepared rGO-Ni at various reaction times, and EDS results illustrate 20 min reaction time leads to a better reduction of GO to rGO.

As prepared, rGO-coated composite confirms its own superhydrophobic nature, and interestingly, the additional rGO-Ni-coated sample resulted in increasing superhydrophobicity. Thus, the presence of Ni created spikes more evenly distributed or periodic spikes on the surface, providing unique interaction with water droplets to form superhydrophobic surfaces.

## Figures and Tables

**Table 1:** Decomposition results of graphite.

Peak	Peak Type	FWHM	Intensity	Center	Area (%)	I_D_/I_G_
D*	Lorentz	44.10074	2.30117	1263.187	0.37276	0.0055
D**	Gauss	88.18919	15.12388	1471.11	3.38514	0.036
D***	Lorentz	20.34075	13.58623	1518.833	1.02809	0.03125
G	Lorentz	25.53374	416.2986	1565.301	39.48637	-
D’	Lorentz	34.40976	32.90883	1603.995	4.19551	0.0792
2D	Lorentz	73.10103	137.5077	2684.201	36.03281	0.33

**Table 2:** Decomposition results of GO and rGO.

	Peak	Peak Type	FWHM	Intensity	Center	Area (%)	I_D_/I_G_
GO	D	Lorentz	103.871	111.7583	1345.573	52.09472	0.87
	D*	Lorentz	76.86495	25.79214	1464.012	9.02568	0.2
	D**	Gauss	36.33935	21.84247	1507.944	2.51529	0.17
	G	Lorentz	72.56196	128.7674	1576.557	29.60897	-
	2D	Lorentz	63.38208	23.66892	2682.37	6.75535	0.18
rGO-Ni 5 min	D	Lorentz	72.36115	400.6227	1338.942	28.3476	0.625
	D*	Gauss	190.0877	208.7136	1488.591	27.05743	0.325
	D**	Lorentz	47.10426	48.40645	1522.598	2.25922	0.075
	G	Lorentz	35.12459	640.2111	1565.578	22.37714	-
	D’	Lorentz	53.94309	316.8547	1606.357	16.91193	0.494
	2D	Lorentz	68.07937	46.27574	2675.5	3.04668	0.072
rGO-Ni 10 min	D	Lorentz	59.55056	94.77432	1341.33	19.9872	0.32
	D*	Lorentz	77.81392	23.73011	1391.398	6.49989	0.078
	D**	Gauss	115.7242	23.72099	1546.179	6.81632	0.078
	G	Lorentz	22.21343	296.4301	1566.151	23.91736	-
	D’	Lorentz	42.80226	68.37753	1599.675	10.54987	0.23
	2D	Lorentz	75.84276	121.4737	2682.816	32.22935	0.41
rGO-Ni 20 min	D	Lorentz	114.0963	261.4571	1353.322	50.3196	1.144
	D*	Lorentz	114.0583	121.8159	1541.898	23.60508	0.53
	G	Gauss	60.28815	228.7064	1599.37	16.4825	-
	G’	Lorentz	72.94592	32.05832	2625.254	3.97089	0.14
	D + D’	Lorentz	128.4541	27.1002	2726.328	5.62193	0.118
rGO-Ni 30 min	D	Lorentz	72.95094	872.99907	1342.89959	39.75877	0.82
	D*	Lorentz	69.28206	84.36227	1412.81349	3.66186	0.079
	D**	Gauss	132.96085	213.42246	1522.65928	12.35659	0.2
	G	Lorentz	36.67842	1061.63651	1575.12468	24.72983	-
	D’	Lorentz	31.03816	321.42136	1608.95909	6.34852	0.3
	2D	Lorentz	88.23703	244.30122	2679.46551	13.14443	0.23

**Table 3:** The EDS of the rGO-Ni.

No.	Element	Before MWI Atomic %	5 min. Atomic %	10 min. Atomic %	20 min. Atomic %	30 min. Atomic %
1	C	61.75	55.96	62.1	64.08	47.34
2	O	32.53	36.86	25.06	30.58	42.11
3	S	3.99	3.7	3.76	2.67	4.03
4	Ni	1.73	3.49	9.06	2.67	6.51
	C/O ratio	1.89	1.52	2.48	2.10	1.12

## Data Availability

The data presented in this study are available on request from the corresponding author.
